# Quantitative Ultrasound Analysis of Muscle Reinnervation Following Anterior Interosseous Nerve‐To‐Ulnar Motor Branch Supercharged End‐To‐Side Transfer Surgery: A Preliminary Study

**DOI:** 10.1002/mus.70407

**Published:** 2026-09-25

**Authors:** Rémy Liechti, Esther Vögelin

**Affiliations:** ^1^ Department of Plastic and Hand Surgery Inselspital, University Hospital of Bern Bern Switzerland

**Keywords:** grayscale analysis, muscle ultrasound, nerve transfer surgery, neuromuscular regeneration, ulnar neuropathy at the elbow (UNE)

## Abstract

**Introduction/Aims:**

Routine assessment of muscle reinnervation primarily relies on clinical and electromyographic evaluation. The aim of this preliminary study was to evaluate sensitivity of quantitative ultrasound analysis for monitoring intrinsic hand muscle recovery following anterior interosseous nerve (AIN)‐to‐ulnar motor branch supercharged end‐to‐side (SETS) transfer.

**Methods:**

Eight patients (five male, three female; mean age 54.5 years, range 10–80) underwent anterior ulnar nerve transposition with AIN‐to‐ulnar motor branch SETS transfer. High‐resolution ultrasound assessed the first dorsal interosseous (FDI) and hypothenar muscles pre‐ and postoperatively using quantitative grayscale and cross‐sectional thickness (CST) analysis. Functional outcomes included key pinch and grip strength.

**Results:**

Preoperatively, affected FDI and hypothenar muscles exhibited significantly higher grayscale and lower CST values compared with the contralateral side. Following surgery, this difference decreased significantly in both muscle groups (FDI: mean difference [MD] −19.9, 95% CI −33.5 to −6.3; *p* = 0.013; hypothenar: MD −21.2, 95% CI −32.1 to −10.3; *p* = 0.006). CST measurements improved in the FDI muscle (MD −7.1 mm, 95% CI −11.3 to −2.9; *p* = 0.009), while there was no significant CST change in the hypothenar muscle (MD −2.3 mm, 95% CI −4.9 to 0.4; *p* = 0.081). There was a significant correlation between FDI muscle grayscale values and key pinch strength (*R* ^2^ = 0.31), whereas hypothenar echogenicity showed no correlation with grip strength (*R* ^2^ = 0.0001).

**Discussion:**

This preliminary study suggests that quantitative ultrasound of intrinsic hand muscles appears sensitive and can detect structural changes during reinnervation following AIN‐to‐ulnar motor branch SETS transfer.

**Abbreviations**
AIN: anterior interosseous nerve
ALS: amyotrophic lateral sclerosis
CST: cross‐sectional thickness
FDI: first dorsal interosseous
JAMAR: JAMAR dynamometer (used for grip strength measurement)
MD: mean difference
ROI: region of interest
SETS: supercharged end‐to‐side
STROBE: strengthening the reporting of observational studies in epidemiology

## 1 Introduction

Recovery of intrinsic hand muscles after surgical decompression and/or anterior ulnar nerve transposition in ulnar neuropathy at the elbow (UNE) is often limited, prompting the use of nerve transfer techniques to improve outcomes [1, 2, 3]. Anterior interosseous nerve (AIN)‐to‐ulnar motor branch supercharged end‐to‐side (SETS) transfer has emerged as a valuable adjunctive procedure for UNE with axonal loss [4]. By providing additional donor axons from the intact AIN to the distal ulnar motor branch while preserving native ulnar nerve continuity, the SETS transfer aims to accelerate reinnervation of intrinsic hand muscles, reduce irreversible denervation atrophy, and improve functional recovery, particularly key pinch strength and claw deformity [5, 6]. A recent systematic review of AIN‐to‐ulnar motor nerve SETS transfers reported favorable outcomes, with approximately 75% achieving Medical Research Council (MRC) grade ≥ 3 recovery in the first dorsal interosseous (FDI) muscle after a mean time to recovery of about 7 months following surgery [7].

Evaluating the extent of muscle denervation and subsequent reinnervation remains challenging. Clinical motor scores and strength assessments provide functional data but lack sensitivity for early or subtle changes [8, 9, 10]. Electrodiagnostic studies, particularly needle electromyography, are established tools for assessing muscle denervation and reinnervation but are invasive, may be poorly tolerated, and are impractical for frequent assessments [11, 12, 13]. Therefore, high‐resolution musculoskeletal ultrasound of both nerve and muscle tissue has gained attention as a valuable imaging modality, owing to its ability to visualize nerve morphology and muscle architecture in real time, even in postoperatively altered soft tissues [14, 15]. Unlike other imaging modalities, real‐time ultrasound enables dynamic assessment of muscle fasciculations and contractions [16]. However, these assessments are predominantly qualitative and are constrained by variability related to both the equipment and the operator. Quantitative grayscale analysis is an imaging method to assess muscle echogenicity, which increases with denervation due to fatty infiltration and fibrosis [16, 17, 18]. Prior studies have shown that increased muscle echogenicity correlates with denervation in conditions like amyotrophic lateral sclerosis (ALS) and common entrapment neuropathies [19, 20, 21]. A recently published study showed that quantitative grayscale analysis of target muscles correlated with electromyographic severity and disease duration in patients with carpal tunnel syndrome and UNE [22]. In a preclinical study, grayscale analysis in muscle ultrasound proved to be a reliable method for assessing motor recovery in a rabbit model of peroneal nerve reconstruction [23]. However, there is a lack of data on the systematic application of this method to track reinnervation changes following nerve transfer procedures in humans.

In this preliminary study, we evaluate grayscale ultrasound changes in patients undergoing end‐to‐side AIN‐to‐ulnar motor branch SETS transfer. We hypothesize that quantitative ultrasound can detect meaningful changes in muscle quality over time and possible muscle regeneration correlates with clinical recovery.

## 2 Methods

The research was conducted in accordance with the Declaration of Helsinki and was approved by the Swiss Ethic Committee (Nr. 2025‐01735). This study was designed according to the Quality Appraisal Checklist for Case Series Studies by The Institute of Health Economics, Alberta, Canada [24]. This article was written in accordance with the Strengthening the Reporting of Observational Studies in Epidemiology (STROBE) guidelines [25].

### 2.1 Study Design and Patient Selection

This retrospective observational study included patients who underwent surgical treatment for UNE at a single tertiary referral center. Eligible patients were those who received AIN‐to‐ulnar motor branch SETS transfer and had both preoperative and postoperative clinical and ultrasound evaluations of the FDI and/or abductor digiti minimi (ADM) muscles. Timepoints of the postoperative ultrasound varied significantly due to the retrospective nature of the study. The indication for an AIN‐to‐ulnar motor branch SETS transfer was established in patients with UNE presenting with electrophysiological abnormalities, including slowing of motor nerve conduction velocity across the elbow and reduced or absent compound muscle action potential (CMAP) amplitudes when recording from the ADM, together with clinical signs of persistent paresthesia, intrinsic muscle weakness, and muscle wasting.

Patients with concomitant neuromuscular disorders were excluded from the study. The study was approved by the institutional ethics committee, and all patients provided informed consent for the use of their clinical and imaging data.

### 2.2 Surgical Procedure

All patients underwent decompression of the ulnar nerve at the elbow followed by anterior intramuscular transposition followed by AIN‐to‐ulnar motor branch SETS transfer according to the technique described by Novack et al. [26]. The AIN was coapted end‐to‐side to the ulnar nerve motor branch using microsurgical technique. Postoperatively, patients followed a standardized rehabilitation protocol emphasizing early mobilization and progressive strengthening under the guidance of certified hand therapists.

### 2.3 Ultrasound Examination

All ultrasound examinations were performed using a high‐resolution linear array transducer (eL17–18.4 MHz) on a clinical musculoskeletal ultrasound system (Philips Affiniti 70G, Philips Ultrasound LLC, Bothell, WA, USA). Machine settings, including gain, depth, and frequency, were kept constant between sessions for each patient to ensure reproducibility across pre‐ and postoperative measurements.

Patients were seated comfortably with the forearm supinated and resting on a flat surface below heart level. Both hands were examined during the same session to enable direct side‐to‐side comparison with exactly the same gain, depth, and frequency settings in order to reduce measurement bias.

Ultrasound assessments were performed on two intrinsic hand muscle groups: FDI and hypothenar muscle group. For each muscle, the transducer was placed perpendicular to the skin surface to obtain a standardized transverse view at the thickest portion of the muscle belly. Care was taken to avoid anisotropy by fine adjustments of probe angle. Light probe pressure was maintained to prevent compression of superficial soft tissues.

Quantitative grayscale analysis relies on extracting pixel‐intensity information from standardized B‐mode ultrasound images. All measurements were obtained using the built‐in grayscale histogram functions available on the ultrasound system, which convert the brightness of each pixel into numerical values on an 8‐bit scale ranging from 0 (pure black) to 255 (pure white). For each muscle, a transverse B‐mode image was captured over the thickest cross‐section of the muscle belly at equal locations on both hands. A region of interest (ROI) was manually placed within the central portion of the muscle, avoiding bone interfaces, tendons, and fascial borders, which can artificially elevate grayscale values. The ROI dimensions were kept constant between examinations, and care was taken to avoid anisotropy by ensuring perpendicular probe orientation. The ultrasound machine's histogram tool was then used to compute the mean pixel intensity within the ROI, providing a quantitative echogenicity score in arbitrary units on the 0–255 scale (Figure S1). Cross‐sectional thickness (CST) was measured in millimeters as the maximal muscle thickness between the superficial and deep fascial boundaries on transverse imaging.

Side‐to‐side comparisons were performed using identically acquired contralateral muscle images to serve as internal controls (Figure S2). This approach minimizes patient‐specific variability related to age, systemic health, body composition, or hydration status and enables more sensitive detection of pathological changes. By pairing grayscale values from the affected and unaffected sides, the analysis further reduces the influence of fixed machine settings and enhances interpretability.

All ultrasound scans were performed by the senior author, a highly trained examiner experienced in musculoskeletal imaging. To minimize inter‐session variability, the same examiner performed both preoperative and postoperative examinations for each patient.

### 2.4 Clinical Assessments

Functional outcomes were evaluated using key pinch strength and grip strength measurements, performed with a standardized pinch gauge and JAMAR dynamometer (Performance Health Supply Inc., Warrenville, IL, USA), respectively. Strength testing was performed on both hands, and values from the affected side were expressed as a percentage of the strength of the unaffected side to minimize inter‐individual variability.

### 2.5 Statistical Analysis

Statistical analyses were conducted using GraphPad Prism software version 10.6.1 (GraphPad Software, Boston, MA, USA). Normality of data distribution was assessed using the Shapiro–Wilk test. For normally distributed paired data, paired *t*‐tests were used to compare preoperative and postoperative side‐to‐side differences in grayscale echogenicity and CST for both muscle groups. To illustrate effect sizes and confidence intervals, results were visualized using estimation plots, which display raw paired data, mean differences (MD), and 95% confidence intervals. Associations between ultrasound parameters and functional measures (key pinch strength, grip strength) were assessed using simple linear regression analysis. Model fit was quantified using the coefficient of determination (*R* ^2^), and statistical significance was set at *p* < 0.05.

## 3 Results

### 3.1 Patient Characteristics

A total of eight patients (five male, three female) with a mean age of 54.5 years (range 10–80 years) underwent AIN‐to‐ulnar motor branch SETS transfer for UNE. Etiologies included traumatic UNE in three patients and idiopathic UNE in five patients. Patient characteristics are displayed in Table 1. Mean follow‐up duration was 23.9 months (range 7–39 months).

**TABLE 1:** Baseline patient characteristics.

Patient	Etiology	Sex	Age (years)	Preop duration of symptoms (months)	Preop ADM CMAP amplitude (mV)	Preop motor velocity (m/s)	Preop key pinch strength (% of contralateral side)	Preop two point discrimination little finger (mm)	Preop pain (yes, no)	Preop Tinel's sign (+/−)
1	Chronic compression	M	65	39	1.0	36.5	70	> 10	No	+
2	Elbow dislocation	F	51	7	0	—	50	7	No	−
3	Chronic compression	M	71	30	2.8	28.5	56	8	No	+
4	Medial epicondyle fracture	F	10	30	2.6	38.0	63	> 10	No	+
5	Chronic compression	M	80	27	3.6	40.2	0	> 10	No	+
6	Chronic compression	M	66	30	0	—	60	3	Yes	+
7	Chronic compression	F	28	62	0.7	47.0	44	4	Yes	+
8	Elbow fracture dislocation	M	65	65	6.0	38.5	19	5	No	+
Mean (SD)			54.5 (23.8)	36.3 (19.1)	2.1 (2.1)	28.6 (18.4)	54.2 (23.9)			

Abbreviations: CMAP, compound muscle action potential; OP, operation; US, ultrasound.

### 3.2 Grayscale Echogenicity Analysis

Baseline and postoperative absolute and normalized grayscale values of the FDI and ADM muscles are given in Table 2. Preoperative ultrasonography demonstrated significantly increased grayscale echogenicity in the affected muscles compared with the contralateral side, reflecting denervation changes. Specifically, the FDI muscle showed a MD of 26.4 (95% CI 12.3–40.5, *p* = 0.005), and the hypothenar muscles exhibited a MD of 32.8 (95% CI 18.9–46.7, *p* = 0.002).

**TABLE 2:** Pre‐ and postoperative absolute and normalized grayscale values per patient.

Patient	Follow‐up for time (months)	FDI				ADM			
		Grayscale absolute values		Normalized to contralateral side		Grayscale absolute values		Normalized to contralateral side	
		Preop	Postop	Preop	Postop	Preop	Postop	Preop	Postop
1	17.0	113.2	86.7	27.2	0.3	76.1	81.2	19.9	4.1
2	24.0	126.0	110.0	23.3	9.8	101.4	77.7	30.3	8.7
3	26.9	134.2	88.5	38.6	10.9	—	142.6	—	29.1
4	38.7	—	97.5	—	15.7	71.4	96.9	35.2	21.7
5	16.0	146.3	158.7	10.0	5.6	126.9	—	16.7	—
6	7.0	111.4	128.3	44.7	6.6	96.9	122.9	44.0	24.9
7	23.0	—	81.1	—	0.0	112.2	96.3	50.5	14.6
8	39.0	102.5	85.6	14.5	5.8	—	68.4	—	1.7
Mean (SD)	23.9 (11.0)	122.3 (16.3)	104.5 (26.9)	26.4 (13.4)	6.8 (5.3)	97.5 (21.1)	98.0 (26.4)	32.8 (13.2)	15.0 (10.6)

Following surgery, echogenicity differences between the affected and contralateral sides decreased significantly in both muscle groups, suggesting structural recovery due to reinnervation. The FDI muscle demonstrated a mean postoperative grayscale reduction of −19.9 (95% CI −33.5 to −6.3, *p* = 0.013), while the hypothenar muscles showed a reduction of −21.2 (95% CI −32.1 to −10.3, *p* = 0.006). Estimation plots summarizing these changes are presented in Figure 1.

**FIGURE 1:**
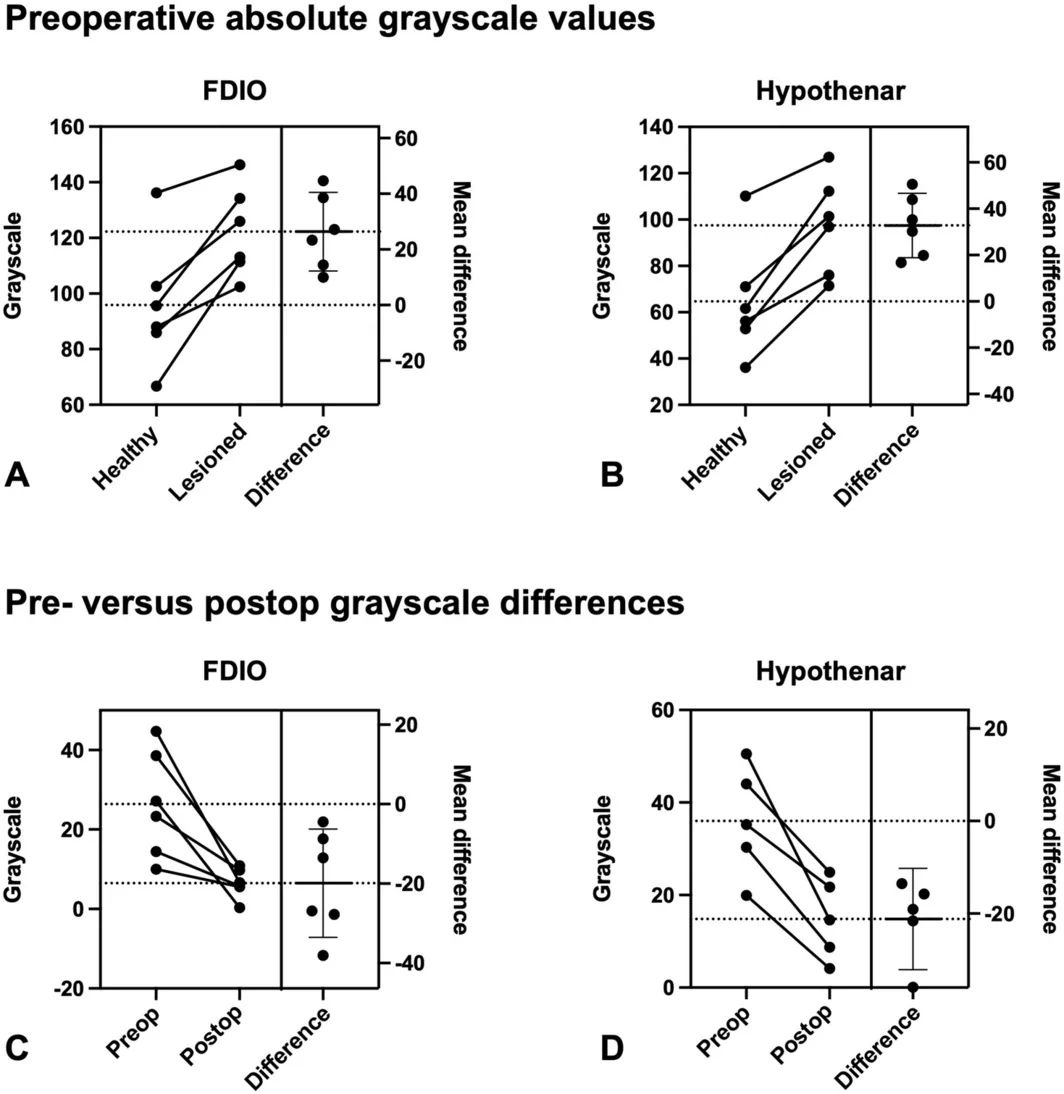
Estimation plots showing side‐to‐side differences in grayscale echogenicity of the first dorsal interosseous (FDI) and hypothenar muscles. Preoperatively, affected muscles exhibited significantly higher echogenicity compared with the contralateral side (A, B). Postoperatively, side‐to‐side differences decreased significantly in both muscle groups (C, D). Raw paired data, mean differences, and 95% confidence intervals are displayed.

### 3.3 CST Measurements

Preoperative CST assessments revealed significant atrophy in the affected muscles relative to the contralateral side. The mean CST difference between sides for the FDI muscle was −8.7 mm (95% CI −11.6 to −5.8, *p* = 0.001), and for the hypothenar muscles −4.7 mm (95% CI −7.0 to −2.4, *p* = 0.003).

Postoperatively, CST side‐to‐side differences decreased significantly in the FDI (MD −7.1, 95% CI −11.3 to −2.9, *p* = 0.009). There was no statistically significant difference in CST in the hypothenar muscles (MD −2.3, 95% CI −4.9 to 0.4, *p* = 0.081). Estimation plots for CST analyses are shown in Figure 2.

**FIGURE 2:**
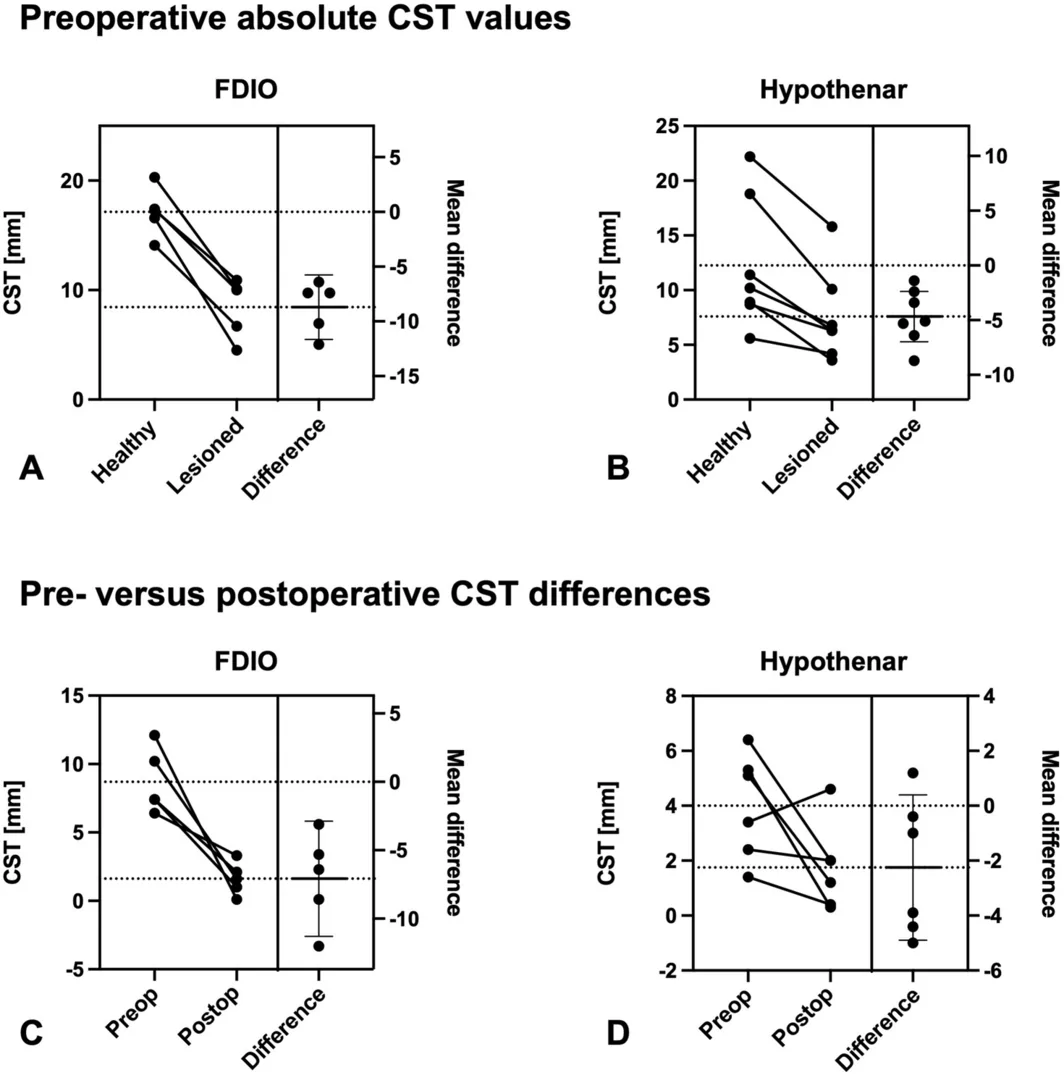
Estimation plots of cross‐sectional thickness (CST) for first dorsal interosseous (FDI) and hypothenar muscles. Preoperative measurements demonstrate significant atrophy in affected muscles compared with the contralateral side (A, B). Postoperative assessment shows a significant decrease of inter‐side difference of first dorsal interosseous (FDI) muscle thickness and no pre‐ to postoperative difference in hypothenar muscles (C, D). Raw paired data, mean differences, and 95% confidence intervals are displayed.

### 3.4 Correlation With Functional Outcomes

Functional recovery was evaluated by correlating muscle ultrasound parameters with clinical strength measures. Absolute grayscale values of the affected FDI muscle of both, pre‐ and postoperative measurements, demonstrated a significant correlation with key pinch strength (*R* ^2^ = 0.31, *p* = 0.049). In contrast, hypothenar grayscale values did not significantly correlate with grip strength (*R* ^2^ = 0.0001, *p* = 0.971). Corresponding scatterplots and regression lines are provided in Figure 3.

**FIGURE 3:**
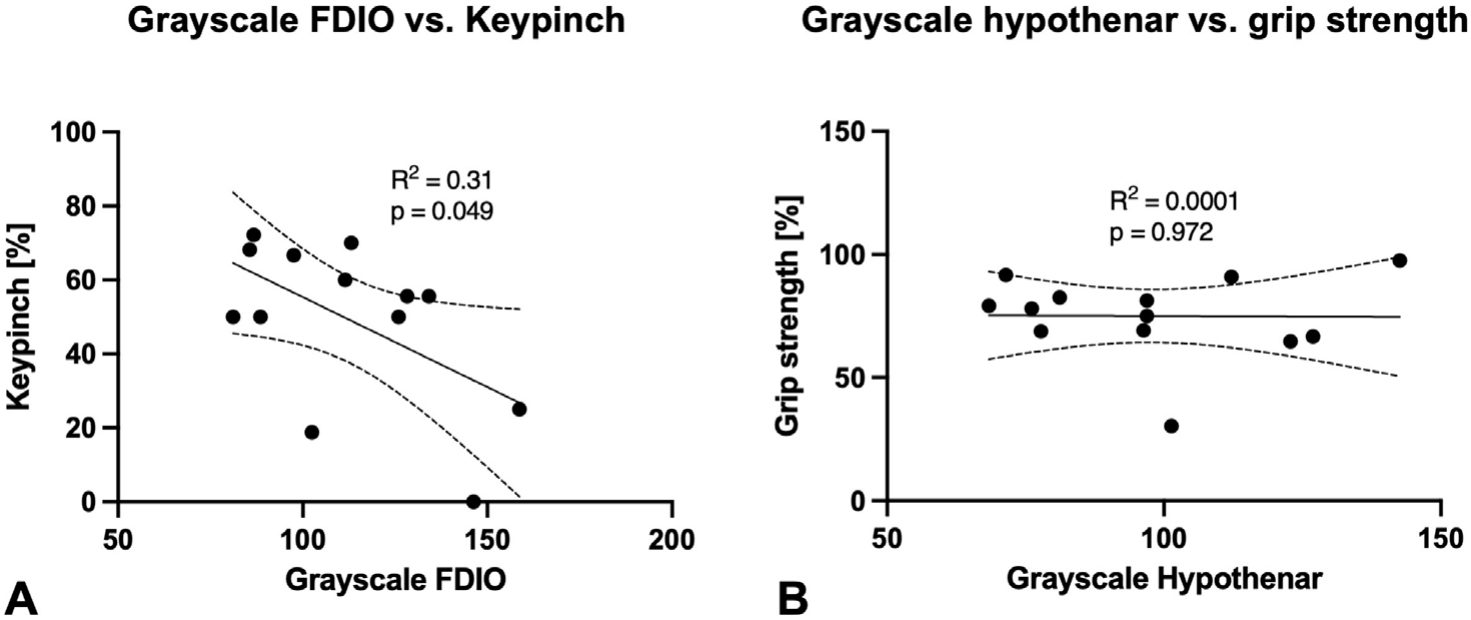
Scatterplots and linear regression analyses depicting the relationship between quantitative grayscale analysis and functional outcomes. First dorsal interosseous (FDI) muscle grayscale values significantly correlated with key pinch strength (A), whereas hypothenar grayscale values did not correlate with grip strength (B). Regression lines and 95% confidence intervals are shown.

## 4 Discussion

### 4.1 Interpretation of Key Findings

The findings of the present study suggest that quantitative grayscale ultrasonography is not only feasible but also capable of detecting meaningful structural changes and correlating with functional recovery following AIN‐to‐ulnar motor branch SETS transfer in patients with UNE. The significant postoperative reductions in muscle echogenicity and improvements in CST observed in intrinsic hand muscles suggest ongoing reinnervation. The association between grayscale values of the FDI muscle and key pinch strength further indicates that ultrasound metrics may serve as meaningful markers of functional recovery. However, it is important to note that this method is only valid when echogenicity and CST values are compared with the contralateral side and assessed through repeated measurements over time.

### 4.2 Echogenicity and CST as Indicators of Denervation and Reinnervation

The markedly elevated preoperative grayscale values recorded in affected muscles align with well‐established ultrasound findings in chronic denervation, in which fatty infiltration and fibrosis lead to increased echogenicity [16, 17, 18]. These results corroborate previous studies in ALS and peripheral entrapment neuropathies, where grayscale ultrasonography effectively identified denervated muscle [19, 20, 21]. These findings are also consistent with the observations of Wang et al., who reported significantly increased grayscale levels in the FDI muscle of patients with UNE and demonstrated stronger associations between FDI echogenicity and electrophysiological measures than for the ADM muscle. In the present study, the FDI likewise exhibited the most pronounced ultrasound changes and was the only muscle in which grayscale values correlated with functional recovery [22]. While Wang et al. focused on chronic denervation, our findings suggest that quantitative grayscale analysis of the FDI may also be sensitive to the structural changes associated with muscle reinnervation following nerve transfer surgery. Following surgery, the significant reduction in echogenicity in both FDI and hypothenar muscles reflects structural improvement consistent with reinnervation. This observation is in line with findings from preclinical models in which grayscale ultrasound reliably tracked reinnervation stages after nerve reconstruction [23]. The sensitivity of grayscale ultrasonography makes it a compelling tool for postoperative monitoring.

CST measurements confirmed substantial preoperative atrophy in intrinsic muscles, consistent with the severity of disease in this cohort. Postoperative improvements in CST were most notable in the FDI muscle, while the hypothenar muscles demonstrated no pre‐ to postoperative difference. Although earlier evidence of reinnervation in the FDI compared with the hypothenar muscles may seem counterintuitive, several studies have reported the same phenomenon, with earlier clinical signs of reinnervation observed in the FDI following AIN‐to‐ulnar motor branch SETS transfer surgery [27, 28]. Since CST generally recovers later in the reinnervation timeline, the findings reinforce the potential of grayscale echogenicity as an earlier indicator of muscle recovery, while CST provides complementary information regarding long‐term muscle volume restoration.

The significant correlation between FDI grayscale echogenicity and key pinch strength illustrates the clinical relevance of quantitative ultrasound assessment. The data suggest that improved muscle structure, as reflected by echogenicity, translates into enhanced functional performance. In contrast, the absence of a relationship between hypothenar echogenicity and grip strength likely reflects the composite nature of grip, which engages numerous extrinsic and intrinsic muscles beyond the hypothenar muscles [29]. This distinction highlights the value of muscle‐specific imaging in evaluating the outcomes of targeted nerve transfers, particularly when global clinical strength measures may fail to isolate the contribution of individual muscles.

### 4.3 Clinical Implications

Muscle ultrasound grayscale measurements are influenced by technical and biological factors, including acquisition settings, operator‐dependent image analysis, and patient characteristics such as age, sex, and body mass index [30]. Consequently, using the contralateral side as an internal control or normalizing values to the contralateral side can reduce the impact of these inter‐individual differences.

Quantitative muscle ultrasound appears to be well suited for integration into postoperative monitoring protocols, offering a noninvasive and reproducible method for assessing reinnervation. The sensitivity of grayscale analysis to changes in muscle structure may allow clinicians to identify delayed or incomplete recovery sooner than is possible with traditional assessments, which in turn could facilitate more timely adjustments to therapy or rehabilitation.

### 4.4 Limitations and Future Directions

Several limitations warrant consideration. The retrospective design and small sample size limit broader generalizability. Because all ultrasound assessments were performed by a single experienced examiner, inter‐observer variability could not be assessed and blinding was not performed. Future studies should involve multiple blinded operators and standardized acquisition protocols to ensure reproducibility. Additionally, the duration of follow‐up varied across patients, introducing heterogeneity in recovery profiles. Prospective studies with predetermined imaging intervals would help clarify the temporal evolution of grayscale and CST changes.

The findings of this pilot study suggest several avenues for further research. Larger and prospectively designed investigations are needed to validate grayscale and CST measurements as standardized biomarkers of reinnervation. In future studies, the abductor pollicis brevis could be evaluated as an ipsilateral control. Combining ultrasonography with electromyography may enable the development of multimodal recovery profiles. There is also potential for incorporating advanced image analysis techniques, such as machine learning‐based texture evaluation, to identify subtle structural changes that may not be captured through grayscale averages alone.

## 5 Conclusion

This study provides preliminary evidence that quantitative ultrasonography is a sensitive and clinically meaningful method for monitoring muscle recovery following AIN‐to‐ulnar motor branch SETS transfer. The observed reductions in echogenicity and improvements in CST correspond with functional recovery, particularly in the FDI muscle. These findings support the integration of quantitative muscle ultrasound into postoperative monitoring and underscore its potential to improve the evaluation and rehabilitation of patients undergoing nerve transfer surgery.

## Author Contributions

**Rémy Liechti:** conceptualization, methodology, investigation, validation, visualization, writing – original draft, writing – review and editing, project administration, formal analysis, software, data curation, resources. **Esther Vögelin:** conceptualization, methodology, supervision, data curation, writing – review and editing, visualization, validation, funding acquisition, resources.

## Funding

The authors have nothing to report.

## Ethics Statement

We confirm that we have read the Journal's position on issues involved in ethical publication and affirm that this report is consistent with those guidelines. The study was approved by the Swiss Ethic Committee (Nr. 2025‐01735).

## Conflicts of Interest

The authors declare no conflicts of interest.

## Supporting information


**Figure S1:** Setting for imaging of the first dorsal interosseous (FDI) muscle next to the first metacarpal bone (MC I) using high‐resolution B‐mode muscle ultrasound. The region of interest (ROI) used for grayscale analysis is indicated, along with the corresponding grayscale histogram and computed mean echogenicity mean values and standard deviations (SD).
**Figure S2:** Representative preoperative B‐mode muscle ultrasound images of a first dorsal interosseous (FDI) muscle on the healthy side (left) and the denervated side (right) with corresponding regions of interest (ROI), grayscale histograms, mean and standard deviation (SD) values.

## Data Availability

The data that support the findings of this study are available from the corresponding author upon reasonable request.
