# Supplementary material for: Quantitative Ultrasound Analysis of Muscle Reinnervation Following Anterior Interosseous Nerve‐To‐Ulnar Motor Branch Supercharged End‐To‐Side Transfer Surgery: A Preliminary Study
Source: Muscle Nerve. 2026 Sep 25;74(6):1587–95. doi: 10.1002/mus.70407 (PMC13749242; doi:10.1002/mus.70407)
Supplement: Supplementary file 1 [file MUS-74-1587-s001.docx]

**Supplementary figure legend**

**Supplementary Figure 1:** Setting for imaging of the first dorsal interosseous (FDI) muscle next to the first metacarpal bone (MC I) using high-resolution B-mode muscle ultrasound. The region of interest (ROI) used for grayscale analysis is indicated, along with the corresponding grayscale histogram and computed mean echogenicity mean values and standard deviations (SD).

**Supplementary Figure 2:** Representative preoperative B-mode muscle ultrasound images of a first dorsal interosseous (FDI) muscle on the healthy side (left) and the denervated side (right) with corresponding regions of interest (ROI), grayscale histograms, mean and standard deviation (SD) values.

**Supplementary Figure 1**

**Supplementary Figure 2**
